# Toward Small CNV Detection in NIPT: A Preliminary Analytical Evaluation of Sequencing Depth and Workflow Configuration

**DOI:** 10.3390/diagnostics16142138

**Published:** 2026-07-08

**Authors:** Pasquale Savarese, Valentina Ronga, Saveria Rivetti, Marianna De Simone, Roberta Coda, Claudia Quartotti, Lidia Copponi, Junlan Wu, Di Wu, Xin Liu, Marco Varelli

**Affiliations:** 1 Istituto Diagnostico Varelli, 80126 Naples, Italy; valentina.ronga@istitutovarelli.it (V.R.); saveria.rivetti@istitutovarelli.it (S.R.); marianna.desimone@istitutovarelli.it (M.D.S.); roberta.coda@istitutovarelli.it (R.C.); claudia.quartotti@istitutovarelli.it (C.Q.); lidia.copponi@istitutovarelli.it (L.C.); marco.varelli@istitutovarelli.it (M.V.); 2 Department of Experimental Medicine, Università degli Studi della Campania Luigi Vanvitelli, 80138 Naples, Italy; 3 Vazyme Red Maple Hi-tech Industry Park, Nanjing 210033, China; wujunlan@vazyme.com (J.W.); wudi02@vazyme.com (D.W.); liuxin01@vazyme.com (X.L.)

**Keywords:** non-invasive prenatal testing, microdeletions, sequencing depth

## Abstract

**Background/Objectives**: Non-invasive prenatal testing (NIPT) performance is influenced by multiple biological and technical factors, including fetal fraction, sequencing depth, library preparation, and bioinformatic processing. This study aimed to compare two NIPT workflow configurations based on Vazyme and Illumina platforms and to evaluate the potential impact of sequencing depth and workflow design on analytical performance. **Methods**: A total of 1000 maternal plasma samples were analyzed using two multiplexing configurations (24-plex for Vazyme and 48-plex for Illumina). A workflow-specific reference baseline was constructed using 700 clinically low-risk samples presumed to be euploid to optimize normalization procedures and CNV detection. In addition, a synthetic 22q11.2 deletion control was evaluated across different fetal fraction levels to assess analytical feasibility for small CNV detection. **Results**: The 24-plex workflow generated a higher number of reads per sample (~18M vs. ~9M) and showed reduced analytical variability, particularly in low fetal-fraction samples. Failure rates were lower in the 24-plex workflow (<1% vs. >2.5%). Baseline optimization improved normalization and signal stability. The synthetic 22q11.2 deletion control (~2.5–3 Mb) was consistently detected at fetal fractions ≥6%, supporting the analytical feasibility of small CNV detection under controlled experimental conditions. **Conclusions**: Higher sequencing depth combined with robust baseline construction may improve NIPT analytical performance and enhance sensitivity for subchromosomal CNV detection. However, the present study should be regarded as a preliminary analytical feasibility study rather than a clinical validation study. Additional prospective investigations including clinically confirmed CNV cases are required to establish the true clinical performance of this approach.

## 1. Introduction

Non-invasive prenatal testing (NIPT) has transformed prenatal screening by enabling the detection of fetal chromosomal abnormalities through analysis of circulating cell-free DNA (cfDNA) in maternal plasma. Since the discovery of fetal cfDNA in maternal [1] plasma, massively parallel sequencing technologies have enabled reliable detection of common autosomal trisomies such as trisomy 21, 18, and 13 [2,3,4]. In recent years, attention has shifted toward expanding NIPT applications beyond whole-chromosome aneuploidies. In fact, recent studies have demonstrated that low-coverage whole-genome sequencing can also enable detection of clinically relevant sub-chromosomal copy number variations when supported by appropriate normalization strategies and reference datasets [5,6]. These applications require improved sequencing depth and robust statistical models to distinguish small CNVs from background noise.

Compared with traditional serum marker-based screening tests, NIPT offers significantly higher sensitivity and specificity, reducing false-positive rates and consequently decreasing the number of unnecessary invasive procedures [4,7]. Its clinical adoption is now global, and major scientific societies recommend offering NIPT to all pregnant women regardless of pre-test risk [8]. However, NIPT is a screening test which depends on several biological and technical factors, including the fetal fraction (FF), sequencing depth and bioinformatic normalization strategies. Low fetal fraction, often associated with early gestational age, high maternal body mass index, or placental dysfunction, remains one of the most important biological factors influencing NIPT accuracy and is associated with increased rates of inconclusive or borderline results [9].

In recent years, several commercial and research platforms have emerged, each characterized by specific workflows, sequencing strategies, and bioinformatic algorithms. Major sequencing companies such as Illumina, Thermo Fisher, and BGI provide integrated NIPT solutions, whereas other manufacturers, including Vazyme, Roche, and Agilent, offer modular library preparation kits adaptable to different sequencing instruments. These differences among commercial kits can significantly impact the overall cost of the test and vary in terms of throughput capacity and bioinformatic analysis approaches, ultimately influencing operational efficiency, sequencing depth, and analytical robustness.

Comparative studies have shown that the choice of library preparation chemistry, batch size, and amplification or clustering efficiency can significantly affect read distribution and z-score stability, particularly in samples with low FF. Sequencing depth has been identified as one of the key determinants of analytical performance in NIPT, particularly when detecting subtle chromosomal imbalances or samples with low fetal fraction [10].

With the evolution of NIPT technologies, research has expanded beyond whole-chromosome aneuploidies to include more complex targets such as microdeletions, microduplications, and monogenic disorders [6,11,12]. However, these applications require greater sequencing depth, more accurate estimation of fetal fraction, and advanced statistical models to distinguish small copy number variations (CNVs) from background noise. Therefore, optimization of workflow parameters—including plasma input volume, library complexity, and reads per sample—remains central to improving the reliability and clinical utility of NIPT across a wide range of indications and populations.

In this study, we compared two different NIPT workflows based on the Vazyme and Illumina platforms, respectively, characterized by different processing configurations (24-sample batches for Vazyme vs. 48-sample batches for Illumina). A total of 1000 samples were processed allowing a direct comparative evaluation of technical and analytical performance. Of the 1000 samples analyzed with the Vazyme method, 700 were selected for the construction of a reference baseline used to optimize normalization procedures and CNV detection.

**Figure 1:**
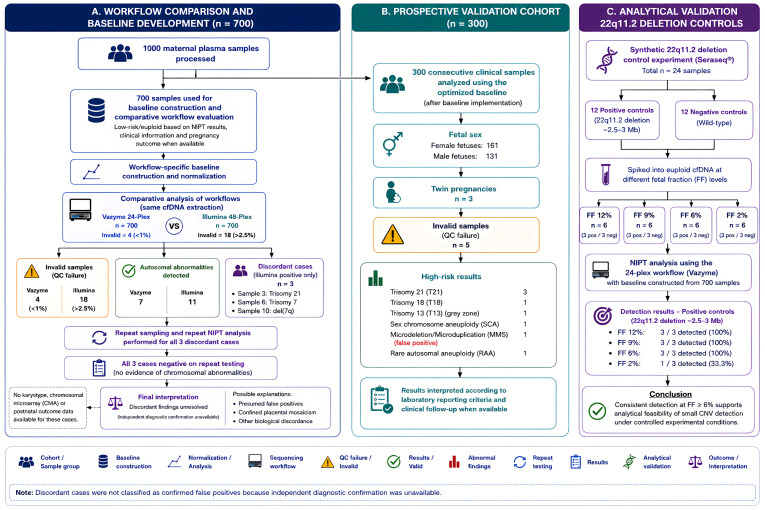
Overview of study design and validation strategy. The study consisted of three complementary phases. First, 700 maternal plasma samples were used for workflow-specific baseline construction and direct comparison between the Vazyme 24-plex and Illumina 48-plex NIPT workflows. Analytical performance, invalid rates, chromosomal abnormalities, and discordant cases were evaluated. Discordant cases underwent repeat sampling and repeat NIPT analysis; however, independent diagnostic confirmation was not available. Second, an independent prospective cohort of 300 consecutive clinical samples was analyzed after implementation of the optimized baseline to assess real-world clinical performance. Third, analytical feasibility of subchromosomal CNV detection was evaluated using synthetic 22q11.2 deletion reference materials tested at different fetal-fraction equivalents. This figure summarizes sample inclusion, baseline development, comparative workflow evaluation, repeat testing procedures, and analytical validation experiments.

Particular attention was given to the relationship between sequencing depth, workflow configuration, and analytical stability. The objective of this study was not to isolate sequencing depth as an independent experimental variable but rather to evaluate the performance of two real-world NIPT workflow configurations characterized by substantially different per-sample sequencing depths. Therefore, the findings should be interpreted within the broader context of workflow-specific technical and bioinformatic characteristics. An overview of the study design and validation strategy is shown in Figure 1.

## 2. Materials and Methods

**Figure 2:**
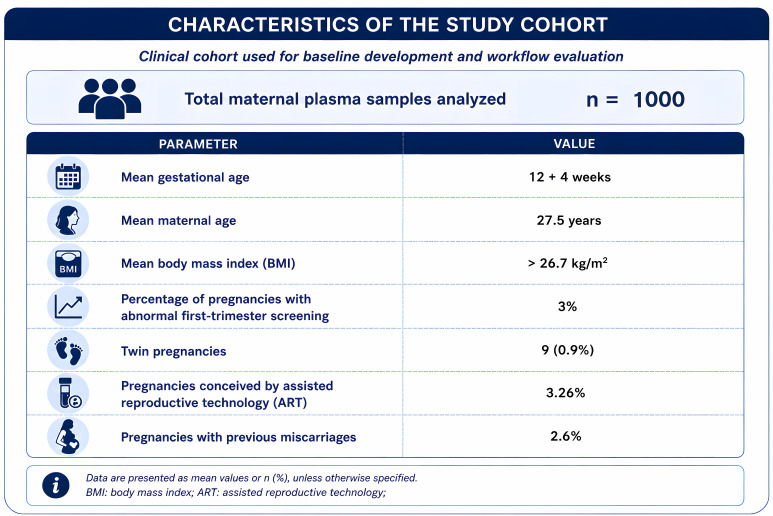
Demographic and clinical characteristics of the study cohort. A total of 1000 maternal plasma samples were included in the study. The cohort was characterized by a mean gestational age of 12 weeks + 4 days, a mean maternal age of 27.5 years, and a mean maternal BMI above 26.7 kg/m^2^. Twin pregnancies accounted for 0.9% of cases (*n* = 9), pregnancies conceived through assisted reproductive technology (ART) for 3.26%, and pregnancies with a history of previous miscarriage for 2.6%. Abnormal first-trimester screening results were reported in 3% of pregnancies.

A total of 1000 maternal plasma samples were processed using two distinct NIPT workflows to enable a direct comparative evaluation of technical and analytical performance. The demographic and clinical characteristics of the study cohort are presented in Figure 2. Circulating cfDNA was extracted according to standardized pre-analytical procedures. Library preparation and sequencing were performed using two different platforms: a Vazyme-based workflow in a 24-plex configuration and an Illumina VeriSeq NIPT workflow in a 48-plex configuration.

### 2.1. DNA Isolation

For extraction and isolation of circulating cell-free DNA (cfDNA), a total of 10 mL of peripheral blood was collected from pregnant women using Streck blood collection tubes. Blood samples were centrifuged at 1600× *g* for 10 min at 4 °C to separate plasma from peripheral blood cells.

### 2.2. Library Preparation—Vazyme Workflow

For the Vazyme workflow, libraries were prepared using the VAHTS^®^ NIPT Library Preparation Kit (Vazyme Biotech, Nanjing, China) in combination with single-index adapters (N805-PB2). The protocol includes end-repair, A-tailing, adapter ligation, and low-cycle PCR amplification, and is optimized for low cfDNA input (typically 5–20 ng), preserving the fragment size distribution (~140–170 bp) and minimizing GC-related bias.

Libraries were prepared on the VNL96 Automated Workstation (Vazyme), quantified using the Equalbit 1× dsDNA HS Assay Kit (Vazyme) on a SpectraMax Gemini XPS microplate reader (Molecular Devices, San Jose, CA, USA), and pooled in batches of 24 samples (24-plex) to maximize per-sample sequencing depth. Sequencing was performed on a NextSeq 550Dx system (Illumina, San Jose, CA, USA) using High Output 75-cycle kits (single-end 1 × 75 bp), with a mean target of >20 million raw reads per sample.

### 2.3. Library Preparation—Illumina Workflow

For the Illumina workflow, maternal plasma processing was performed using a pipeline including automated plasma separation, cfDNA extraction and isolation, library preparation with the VeriSeq NIPT Solution v2 (Microlab STAR, Illumina, San Diego, CA, USA), and shallow whole-genome sequencing (sWGS) on a NextSeq 550Dx platform (Illumina), according to the VeriSeq NIPT Solution v2 package insert (last accessed 1 July 2024) [13].

DNA concentration and purity were assessed by spectrophotometry and fluorometry. Samples were pooled in batches of 48 (48-plex) and sequenced on the NextSeq 550Dx using High Output 75-cycle flow cells, with a target depth of approximately 8–10 million reads per sample, in accordance with the manufacturer’s specifications for aneuploidy screening.

Additional technical details regarding the comparison of the two workflows are reported in the Supplementary Materials.

### 2.4. Illumina Bioinformatic Analysis

Bioinformatic analysis of sequencing data generated by the Illumina workflow was performed using the VeriSeq NIPT CE-IVD pipeline (software version 2.3.0; assay version 1.1.0), compliant with European diagnostic requirements.

The pipeline includes the following steps:

- demultiplexing of sequencing reads
- filtering of low-quality reads
- alignment to the human reference genome (GRCh37/hg19)
- genome-wide coverage normalization
- calculation of chromosome-specific statistical scores.

Interpretation of autosomal and sex chromosome aneuploidies is based on proprietary clinically validated statistical models, which automatically classify samples as high-risk or low-risk according to manufacturer-defined thresholds.

The workflow also integrates quality control metrics, including:

- estimated fetal fraction
- GC content distribution
- duplication rate
- coverage uniformity

These parameters are used to assess data quality and to identify non-interpretable samples.

### 2.5. Vazyme Bioinformatic Analysis

#### 2.5.1. Data Preprocessing and Quality Control

Raw sequencing reads in FASTQ format were processed using fastp (v1.0.1) for adapter removal and quality filtering. Bases with a Phred quality score below Q20 were trimmed from the 3′ end of each read, and reads shorter than 35 bp after trimming were discarded. Reads containing more than 40% low-quality bases or more than five ambiguous nucleotides (N) were excluded from further analysis to ensure high-quality input for downstream copy-number analysis.

#### 2.5.2. Sequence Alignment and Read Filtering

Filtered reads were aligned to the human reference genome (GRCh37/hg19) using Bowtie (v1.3.1) with stringent alignment parameters (-v 0 -m 1), retaining only uniquely mapped reads without mismatches. PCR duplicates and secondary alignments were removed using SAMtools (v1.20) to minimize technical bias and improve copy-number estimation accuracy.

#### 2.5.3. Genome Binning and Coverage Normalization

The reference genome was partitioned into non-overlapping 100-kb genomic bins using BEDTools (v2.31.1). For each sample, uniquely mapped reads were counted within each genomic bin to generate genome-wide coverage profiles.

To reduce systematic sequencing bias associated with GC content, a LOESS (Locally Estimated Scatterplot Smoothing) regression model was applied. Read counts were normalized according to the relationship between genomic GC content and local sequencing coverage. Subsequently, GC-corrected read counts were normalized against the total autosomal read count to compensate for differences in sequencing depth among samples, thereby minimizing inter-sample variability and allowing direct comparison of chromosome-wide coverage profiles.

**Figure 3:**
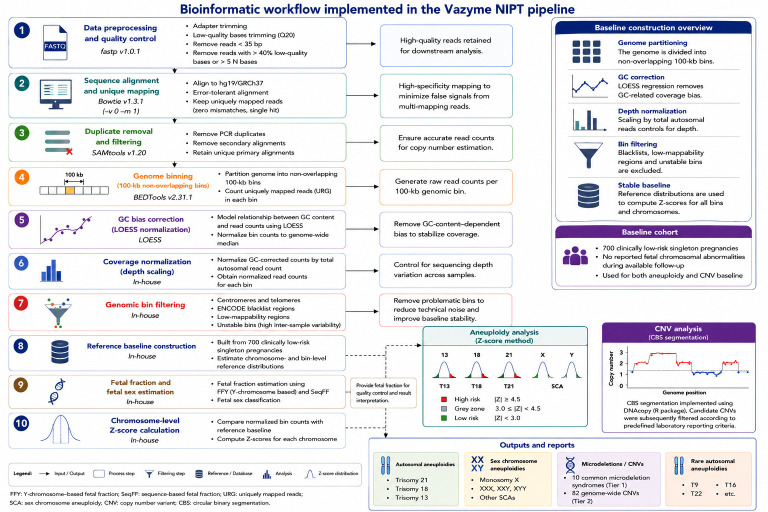
Comprehensive bioinformatic workflow implemented in the Vazyme NIPT pipeline. Raw sequencing reads (FASTQ files) underwent quality filtering and adapter trimming using fastp, followed by unique alignment to the human reference genome (GRCh37/hg19) with Bowtie and removal of PCR duplicates and secondary alignments using SAMtools. Uniquely mapped reads were counted within non-overlapping 100-kb genomic bins generated with BEDTools, followed by GC-content correction using LOESS regression, sequencing-depth normalization, and genomic bin filtering to remove unstable, low mappability, and blacklist regions. A workflow-specific reference baseline, constructed from 700 clinically low-risk singleton pregnancies processed under identical laboratory conditions, was used to estimate chromosome- and bin-level reference distributions for downstream analysis. Fetal fraction was estimated using both the Y chromosome-based approach (FFY) and SeqFF, while chromosome-wide Z-scores were calculated relative to the reference baseline for autosomal and sex chromosome aneuploidy detection. Subchromosomal copy number variants (CNVs) were identified using Circular Binary Segmentation (CBS). The decision engine integrated quality control metrics, fetal fraction, chromosome Z-scores, CNV segmentation results, and predefined reporting thresholds to generate the final clinical interpretation. The pipeline supports the detection of common autosomal aneuploidies (trisomies 13, 18, and 21), sex chromosome aneuploidies, clinically relevant microdeletion and microduplication syndromes, genome-wide subchromosomal CNVs, and rare autosomal aneuploidies.

Bins located within centromeric regions, telomeric regions, ENCODE blacklist regions, and low-mappability genomic intervals were excluded prior to downstream analysis. Additional filtering was performed during baseline construction to remove unstable genomic bins exhibiting excessive inter-sample variability. A schematic overview of the Vazyme bioinformatic pipeline is presented in Figure 3.

#### 2.5.4. Estimation of Fetal DNA Fraction

Fetal fraction (FF) was estimated using two complementary approaches. For pregnancies carrying male fetuses, FF was estimated using the relative representation of Y-chromosome reads compared with male and female reference cohorts (FFY method). For all samples, FF was independently estimated using SeqFF (v1.0), which combines principal component analysis and elastic-net regression applied to autosomal read distributions. Samples with fetal fraction below 4% were considered unsuitable for reliable interpretation and were classified as inconclusive.

#### 2.5.5. Baseline Construction

A workflow-specific reference baseline was constructed using 700 clinically low-risk singleton pregnancies processed with the same laboratory workflow, sequencing platform, and bioinformatic pipeline used for the study samples. Because invasive prenatal diagnosis is not routinely performed in low-risk pregnancies, the majority of baseline samples were not confirmed by karyotyping or chromosomal microarray analysis (CMA). Samples were included in the baseline when classified as low risk by NIPT, with fetal fraction ≥ 4%, absence of reported chromosomal abnormalities, Q30 ≥ 85%, duplication rate < 35%, and acceptable genome-wide coverage uniformity. In addition, no subsequent communication of fetal chromosomal abnormalities, major structural malformations, or adverse pregnancy outcomes was received from the referring clinicians. Therefore, the baseline cohort should be considered a clinically low-risk reference population presumed to be euploid rather than a cohort of universally cytogenetically confirmed euploid pregnancies.

**Figure 4:**
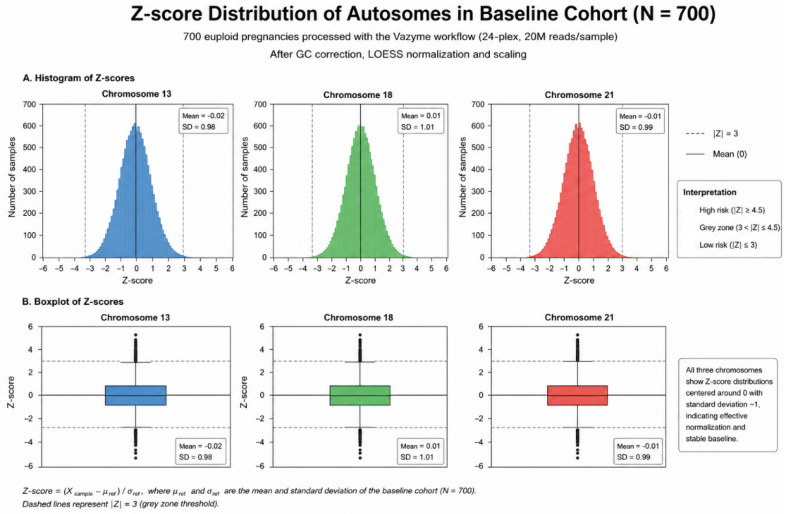
Distribution of chromosome 13, 18, and 21 Z-scores in the 700-sample baseline cohort after normalization. Histograms and corresponding boxplots illustrate the distribution of normalized Z-scores for chromosomes 13, 18, and 21 following GC-content correction, LOESS normalization, and baseline scaling. Colors identify the three chromosomes analyzed (blue: chromosome 13; green: chromosome 18; orange: chromosome 21). All three chromosomes exhibit approximately Gaussian distributions centered around zero, with medians close to zero and standard deviations close to one. The absence of systematic bias or excessive dispersion indicates effective normalization and robust estimation of chromosome-specific variability. These results demonstrate the stability and reliability of the reference baseline, supporting its use for accurate downstream detection of fetal autosomal aneuploidies.

**Figure 5:**
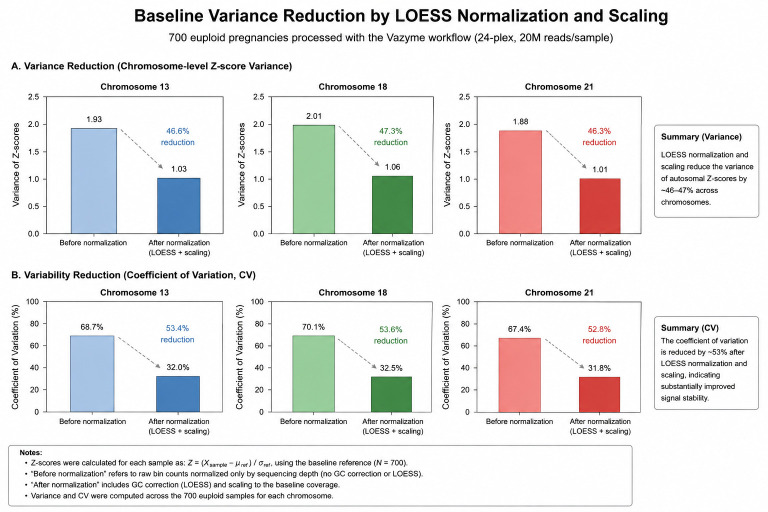
Reduction in baseline variance following LOESS normalization and coverage scaling. Comparison of chromosome-level variance and coefficient of variation (CV) before and after normalization procedures. LOESS-based GC correction and sequencing-depth normalization substantially reduced technical variability across the baseline cohort, improving signal stability and increasing the robustness of Z-score estimation for aneuploidy and CNV analysis.

To improve baseline stability, samples exhibiting excessive coverage variability, defined as a coefficient of variation exceeding three standard deviations from the cohort mean, were excluded. Genomic bins showing excessive inter-sample fluctuation across the reference cohort were likewise identified as unstable and removed from the final reference model. The resulting baseline was used to estimate chromosome-level and bin-level reference distributions, including mean coverage and variance parameters required for downstream Z-score calculation. The large baseline cohort enabled robust estimation of genomic variance, improved signal-to-noise ratio, reduced inter-run variability, and increased analytical stability for the detection of low-amplitude copy-number alterations. Because reference baseline performance is highly dependent on pre-analytical procedures, library preparation chemistry, sequencing platform, sequencing conditions, and bioinformatic processing, the baseline was generated exclusively from samples processed under identical laboratory conditions. Any substantial modification of these analytical variables would require reconstruction and validation of a new workflow-specific reference baseline before routine clinical implementation. The effectiveness of baseline normalization was evaluated by assessing chromosome-level Z-score distributions and variance reduction across the baseline cohort. The distribution of chromosome-specific Z-scores obtained after baseline normalization is shown in Figure 4, whereas the corresponding reduction in chromosome-level variance and coefficient of variation achieved by LOESS normalization and baseline scaling is illustrated in Figure 5.

#### 2.5.6. Detection of Chromosomal Aneuploidies

Chromosomal aneuploidies were detected using a Z-score-based approach comparing normalized chromosome-wide read counts with the corresponding reference baseline distributions. Autosomal trisomies (T13, T18, and T21) were classified as high risk when |Z| ≥ 4.5. Samples with Z-scores between 3.0 and 4.5 were classified as belonging to a grey zone and were subjected to additional review. Sex chromosome aneuploidies (SCAs) were evaluated through combined assessment of X- and Y-chromosome signals relative to sex-specific reference distributions.

#### 2.5.7. Detection of Subchromosomal Copy Number Variants

Subchromosomal copy number variants (CNVs) were identified using an in-house algorithm based on Circular Binary Segmentation (CBS) implemented through the DNAcopy R package. Chromosome-wide normalized bin-level Z-score profiles were segmented into contiguous genomic regions displaying statistically homogeneous copy-number signals. The CBS algorithm recursively searched for statistically significant change-points along each chromosome, partitioning adjacent genomic bins into genomic regions sharing homogeneous copy-number characteristics. Only statistically significant breakpoints were retained, and candidate CNVs were subsequently filtered according to predefined quality criteria, including minimum segment size, minimum supporting bin number, fetal fraction, local genomic mappability, and signal-to-noise ratio. Detected CNVs were classified into two categories:

Tier 1—Clinically Relevant Microdeletion and Microduplication Syndromes. Targeted genomic regions associated with well-characterized syndromes (including 22q11.2 deletion syndrome, 5p deletion syndrome, and 1p36 deletion syndrome) were reported when the segment size exceeded 2.5 Mb and the segment Z-score exceeded predefined significance thresholds. Tier 2—Genome-wide Subchromosomal CNVs. Genome-wide CNVs larger than 7 Mb showing significant copy-number deviation were also reported. Potential maternal CNVs were evaluated separately to distinguish maternal genomic variation from fetal abnormalities whenever possible.

**Figure 6:**
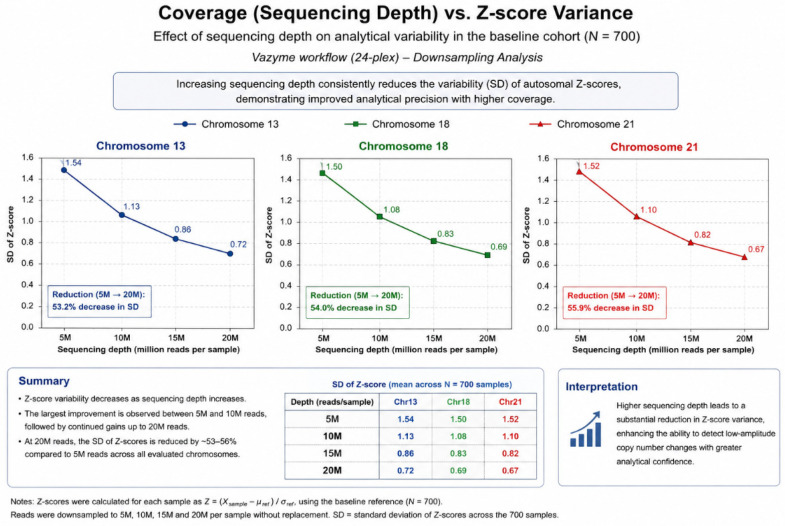
Relationship between sequencing depth and Z-score variability. Representative analysis illustrating the effect of increasing sequencing depth on chromosome-level Z-score standard deviation. Higher sequencing depth was associated with progressive reduction in analytical variability, resulting in improved signal-to-noise ratio and enhanced theoretical sensitivity for the detection of low-amplitude chromosomal abnormalities and subchromosomal copy-number variants.

Because sequencing depth is a major determinant of signal-to-noise ratio in low-coverage whole-genome sequencing, the relationship between read depth and Z-score variability was further evaluated through depth-dependent variance analysis (Figure 6). The underlying assumption of the CBS approach is that a true fetal copy-number alteration produces a coordinated shift across multiple adjacent genomic bins rather than isolated stochastic fluctuations. By aggregating weak but consistent signals across contiguous genomic regions, CBS improves the signal-to-noise ratio and enhances the analytical sensitivity for detecting low-amplitude subchromosomal abnormalities.

#### 2.5.8. Result Interpretation

Samples were interpreted according to predefined laboratory reporting criteria. Quality control: Samples with fetal fraction below 4% were classified as inconclusive, and repeat blood sampling was recommended.

Autosomal aneuploidies:

- High risk: |Z| ≥ 4.5
- Grey zone: 3.0 ≤ |Z| < 4.5
- Low risk: |Z| < 3.0

Sex chromosome aneuploidies: Interpretation was based on combined evaluation of X- and Y-chromosome signals relative to the reference baseline. Copy number variants: CNVs were reported when segmentation analysis identified statistically significant copy-number deviations exceeding predefined laboratory thresholds. All high-risk findings were recommended for confirmation by invasive prenatal diagnosis, including amniocentesis or chorionic villus sampling followed by karyotyping and/or chromosomal microarray analysis (CMA).

### 2.6. Statistical Analysis

Statistical analyses were performed to compare key performance metrics between workflows. Sequencing depth distributions were compared using the Mann–Whitney U test because read counts were not assumed to follow a normal distribution. Failure rates were compared using Fisher’s exact test. Confidence intervals for categorical variables were calculated using the exact Clopper–Pearson method. A two-sided *p*-value < 0.05 was considered statistically significant.

## 3. Results

Firstly, a total of 700 cfDNA samples were analysed both with Vazyme and Illumina workflows. The Vazyme workflow generated a significantly higher number of reads per sample (mean ~18 million) compared with the Illumina workflow (~9 million). The higher read depth improved z-score stability and reduced variability in low fetal fraction samples (Table 1). Formal statistical comparison demonstrated a significantly lower failure rate for the Vazyme workflow compared with the Illumina workflow (4/700 vs. 18/700; Fisher’s exact test, *p* = 0.0041).

Overall, the failure rate was <1% (4/700) for Vazyme and >2.5% (18/700) for Illumina. For Illumina, this value decreased to <2% after repeat sampling and re-analysis, indicating that a substantial proportion of initial failures can be recovered through retesting.

### 3.1. Sequencing Depth and Fetal Fraction

**Figure 7:**
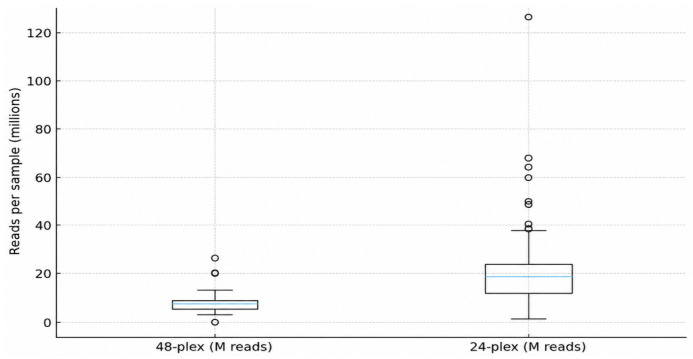
Total reads per sample for the 48-plex and 24-plex workflows. Boxplots indicate the median, interquartile range, whiskers (1.5 × IQR), and outliers.

Vazyme generated a significantly higher sequencing depth (mean 17.97 M reads; median 17.61 M) compared with Illumina (mean 7.32 M reads; median 8.68 M). This difference remained highly significant after formal statistical comparison (Mann–Whitney U test, *p* < 0.0001) (Figure 7). This result is notable given the lower plasma input volume required by Vazyme (0.4 mL vs. 1.0 mL) and the smaller batch size (24 vs. 48 samples), supporting a higher overall workflow efficiency.

**Figure 8:**
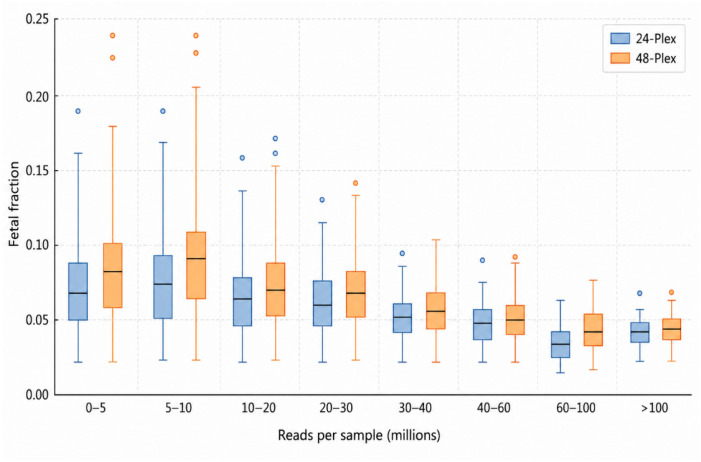
Relationship between read depth and fetal fraction (both platforms).

**Figure 9:**
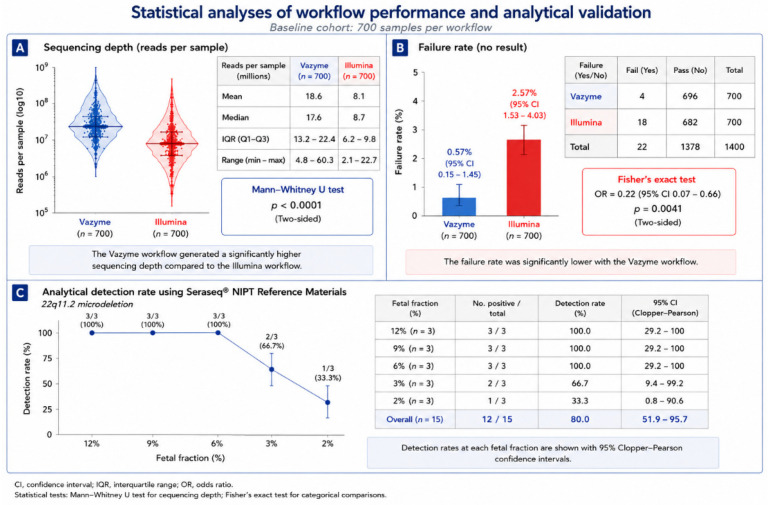
Formal statistical comparison of key workflow performance metrics between the Vazyme and Illumina NIPT workflows. (**A**) Comparison of sequencing depth in the 700-sample baseline cohort. The Vazyme workflow generated significantly higher numbers of reads per sample than the Illumina workflow, as demonstrated by the Mann–Whitney U test (*p* < 0.0001). Mean, median, interquartile range (IQR), and read count ranges are reported for both workflows. (**B**) Comparison of workflow failure rates in the baseline cohort. The Vazyme workflow showed a significantly lower failure rate than the Illumina workflow (4/700 vs. 18/700; Fisher’s exact test, *p* = 0.0041). Error bars represent exact 95% Clopper–Pearson confidence intervals, and the corresponding odds ratio (OR) with 95% confidence interval is reported. (**C**) Analytical performance of the Vazyme microdeletion assay evaluated using Seraseq^®^ NIPT Reference Materials carrying a 22q11.2 microdeletion at different simulated fetal fractions (12%, 9%, 6%, 3%, and 2%). Detection rates progressively decreased with lower fetal fractions, while exact 95% Clopper–Pearson confidence intervals were calculated for each dilution level. These analyses were performed to provide formal statistical support for the comparison of workflow performance and the analytical validation of microdeletion detection.

Correlation between read depth and fetal fraction (FF) was weak for both platforms (Illumina r = 0.05; Vazyme r = −0.10) (Figure 8), indicating that FF is mainly influenced by biological rather than technical factors. The principal statistical analyses supporting the comparison between the two workflows, including sequencing depth, failure rate, and analytical validation using Seraseq^®^ reference materials, are summarized in Figure 9.

### 3.2. Identification of Autosomal Chromosomal Abnormalities

Eight positive autosomal abnormalities were identified using Vazyme and eleven using Illumina, including common trisomies (T21, T18, T13), rare autosomal aneuploidies (RAA), and one deletion > 7 Mb. Details were reported in Table 2.

In three samples (IDs 3, 6, and 10), the abnormality was detected by the Illumina workflow, but not by Vazyme. Evaluation of Z-scores and LLR/MLLR metrics revealed values close to decision thresholds or associated with reduced read depth, conditions known to increase calling variability (Table 3).

These findings suggest that part of the observed discordance may reflect borderline signals or potential false-positive calls, particularly in samples with low FF.

### 3.3. Sampling Repeat and Re-Analysis of the Discordant Samples Between Vazyme and Illumina Workflows

Three samples showed discordant results between the Vazyme and Illumina NIPT workflows, with abnormal findings reported exclusively by the Illumina platform. Specifically, Illumina identified: (I) a trisomy 7 signal in a twin pregnancy (Sample 6), (II) a trisomy 21 signal in a singleton pregnancy (Sample 3), and (III) a 26.8 Mb deletion on chromosome 7 (q21.3–q31.33) (Sample 10). In contrast, all three samples were classified as low risk (WT) by the Vazyme workflow. To clarify these discrepancies, all discordant cases underwent repeat sampling followed by re-analysis using the Illumina workflow. In all three instances, the second analysis returned a negative result, with no evidence of chromosomal abnormalities. Although these findings may suggest that the initial positive calls represented presumed false-positive results, definitive classification is not possible because confirmatory diagnostic procedures such as karyotyping, chromosomal microarray analysis, or postnatal outcome data were not available. Alternative biological explanations, including confined placental mosaicism or other biological sources of NIPT discordance, cannot therefore be excluded.

### 3.4. Sex Chromosome Aneuploidies

The analysis of sex chromosome aneuploidies was not included in the scope of the present study.

### 3.5. Performance of Vazyme NIPT Plus After Baseline Construction

To evaluate the analytical sensitivity of the CNV detection algorithm for small microdeletions, a single 24-plex sequencing run was performed including 12 wild-type (WT) plasma samples and 12 samples spiked with a commercially available 22q11.2 deletion reference material (Seraseq 22q11 Male—Matched Reference Material). The positive control was diluted into plasma from a non-pregnant female at fetal-fraction equivalents of 12%, 9%, 6%, and 2%, with triplicate testing for each dilution. All WT samples were correctly classified as negative. The 22q11.2 microdeletion was detected in all replicates at 12%, 9%, and 6% fetal fraction, showing highly negative z-scores (−12.59 to −9.12) and high genomic overlap (65.5–100%). At 2% fetal fraction, one of three replicates remained positive (Z = −7.37; overlap = 79.93%), whereas two replicates were not called. These findings demonstrate the analytical feasibility of detecting a synthetic ~2.5–3.0 Mb 22q11.2 deletion under controlled experimental conditions. However, these results should not be interpreted as evidence of clinical sensitivity or specificity for CNVs below 3 Mb because synthetic reference materials do not fully reproduce the biological complexity of circulating cell-free DNA in clinical pregnancies (Table 4).

The following table (Table 5) refers exclusively to samples processed with the Vazyme NIPT Plus workflow using the updated CNV algorithm. Following the 24-sample run performed with the 22q11.2 positive control, a total of 300 clinical samples were subsequently analyzed with the new Plus algorithm after baseline implementation.

The table reports sample distribution, invalid rate, twin pregnancies, and high-risk results for common trisomies (T13, T18, T21), sex chromosome aneuploidies (SCA), microdeletions/microduplications (MMS), and rare autosomal aneuploidies (RAA). Grey zone and presumed false-positive cases are indicated where applicable.

Among the 300 samples analyzed, fetal sex distribution and invalid sample rate were consistent with those observed in the main dataset. High-risk results mainly involved common trisomies, with one SCA case and one MMS case subsequently classified as presumed false positive after repeat testing (Table 6).

Overall, after baseline construction, the Vazyme NIPT Plus workflow demonstrated:

- good analytical stability,
- low failure rate,
- reliable detection of common trisomies and RAAs,
- improved performance in samples with low FF.

These data support the use of Vazyme as a reliable platform for clinical NIPT applications, particularly when higher sequencing depth is required for autosomal abnormality detection and potentially small CNV analysis.

Study Limitations: The present study has several limitations. First, the comparison involved two complete workflow configurations that differed not only in sequencing depth but also in library preparation chemistry, sequencing strategy, normalization procedures, and bioinformatic analysis. Therefore, the specific contribution of sequencing depth cannot be isolated from other workflow-related variables. Second, systematic confirmatory diagnostic testing was not available for all positive and discordant cases. Consequently, the true clinical performance of the evaluated workflows cannot be definitively established. Third, the CNV analysis relied primarily on synthetic reference materials and a limited number of clinical observations. Therefore, the reported findings should be interpreted as evidence of analytical feasibility rather than clinical validation. Finally, potential sources of biological discordance, including confined placental mosaicism, maternal CNVs, and vanishing twin phenomena, could not be systematically evaluated.

## 4. Discussion

**Figure 10:**
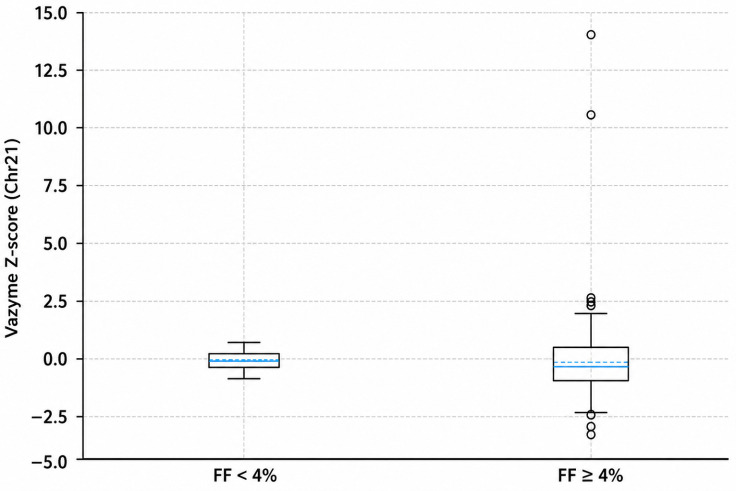
Vazyme chromosome 21 z-score dispersion by fetal fraction group. The blue line represents the mean Z-score.

Recent studies have expanded the scope of non-invasive prenatal testing (NIPT) beyond common aneuploidies toward genome-wide detection of copy number variants (CNVs), emphasizing the critical role of sequencing depth and robust bioinformatic normalization strategies [5,11]. In this context, our findings provide further evidence that increasing per-sample sequencing depth significantly enhances analytical stability and improves the detection of both chromosomal and sub-chromosomal abnormalities [10]. Higher read depth was associated with reduced stochastic variability and improved separation between euploid and aneuploid distributions [10,14]. This effect was particularly evident in low fetal fraction (FF) samples, where analytical noise is typically higher (Figure 10). Notably, in samples with FF < 4%, the variance of chromosome 21 z-scores was markedly lower compared to the overall population (0.155 vs. 1.738), indicating improved metric stability under challenging biological conditions. This should be important to avoid false positive results, such as for the cases of trisomy 21, trisomy 7 and the chromosome 7 deletion case. These results support the concept that sequencing depth can partially compensate for unfavourable sample characteristics by stabilizing statistical outputs. Direct comparison of chromosomal scores between workflows was not feasible due to fundamental methodological differences, as the Illumina VeriSeq pipeline relies on proprietary likelihood-based models (LLR), whereas the Vazyme workflow employs a z-score–based approach. Therefore, cross-platform evaluation was performed using clinical interpretation and performance metrics. A representative discordant case (sample 3), initially classified as high risk for trisomy 21 by Illumina but low risk by Vazyme, was subsequently resolved as normal upon repeat sampling, suggesting a discordant finding potentially associated with low fetal fraction and increased analytical variability, although the absence of independent diagnostic confirmation prevents definitive classification.

The adoption of a 24-plex configuration in the Vazyme workflow was driven by the need to maximize per-sample read depth while maintaining operational flexibility. In parallel, the construction of a large euploid baseline proved essential for accurate CNV detection. A robust reference model enables effective normalization of technical and biological variability, including GC bias, uneven genomic coverage, and batch-specific noise. This normalization improves z-score estimation and enhances the signal-to-noise ratio, which is particularly critical for detecting small CNVs whose amplitude may overlap with physiological cfDNA fluctuations. Importantly, the combination of increased sequencing depth and a large baseline significantly improved the detection of sub-chromosomal CNVs [15,16]. Validation experiments using a synthetic 22q11.2 deletion control demonstrated reliable detection of a ~2.5–3 Mb microdeletion at fetal fractions ≥ 6%. This finding highlights the importance of both sequencing depth and normalization strategies when extending NIPT applications beyond whole-chromosome aneuploidies to clinically relevant microdeletions [12,17,18]. Further improvements were observed following the implementation of an expanded baseline derived from a large cohort of clinical samples. This approach reduced background variability and enhanced computational robustness, particularly for CNVs smaller than 3 Mb, where the signal-to-noise ratio is inherently limited. These results reinforce the importance of dataset size and quality in the development of reliable CNV detection pipelines. From a biological perspective, fetal fraction remains a key determinant of NIPT performance [9]. Factors such as gestational age, maternal body mass index (BMI), anticoagulant therapy, and autoimmune conditions are known to influence FF. While FF showed limited correlation with sequencing read count, confirming its predominantly biological nature, increased sequencing depth effectively mitigated stochastic noise and improved classification in borderline cases. From a clinical and operational standpoint, these findings have several implications. Increasing per-sample sequencing depth is expected to reduce the rate of inconclusive results and the need for redraws, particularly in early gestation and in pregnancies with elevated maternal BMI. In addition, run configuration and batch size should be optimized to ensure sufficient read allocation per sample, especially near reporting thresholds. Laboratories handling low plasma input volumes may benefit from protocols that maximize library complexity and clustering efficiency. Finally, quality control frameworks should incorporate performance monitoring in low-FF samples and in clinically relevant CNV regions, as these represent sensitive indicators of overall assay robustness. Overall, this study demonstrates that the combined optimization of sequencing depth, library preparation efficiency, and baseline size represents a key strategy for advancing NIPT from a test primarily focused on common trisomies toward a broader screening platform capable of reliably detecting clinically relevant CNVs.

This study has several limitations. First, the comparison between workflows was based on different analytical frameworks, with z-score-based metrics available for the Vazyme pipeline and proprietary likelihood-based metrics used in the Illumina workflow, limiting direct statistical comparability across platforms. Second, confirmatory clinical outcomes (e.g., karyotyping or chromosomal microarray analysis) were not systematically available for all cases, and therefore the clinical sensitivity and specificity of CNV detection could not be fully assessed. Third, the use of a 24-plex versus 48-plex configuration introduces an inherent difference in per-sample sequencing depth, which may contribute to the observed performance differences between workflows. Finally, batch-level variables, including flow cell loading, reagent lot variability, and operator-dependent factors, were not explicitly modeled and may have contributed to residual technical variability.

## 5. Conclusions

Per-sample sequencing depth and workflow configuration represent important determinants of NIPT analytical performance. In this study, the 24-plex workflow was associated with higher read depth, lower analytical variability, and improved stability of chromosomal z-scores, particularly in samples with low fetal fraction. Beyond sequencing depth alone, our findings highlight the importance of workflow-specific optimization strategies, including baseline construction and normalization procedures, in reducing background noise and improving signal consistency across samples. The development of a dedicated reference baseline using a large cohort of low-risk pregnancies proved to be a critical component of the analytical framework, contributing to improved normalization, reduced variability, and enhanced confidence in chromosomal and subchromosomal event detection. These observations suggest that baseline quality may represent an underappreciated factor influencing NIPT performance and should be considered alongside sequencing depth when optimizing workflow design. The synthetic 22q11.2 deletion experiment demonstrated the analytical feasibility of detecting small CNVs under controlled experimental conditions, with consistent detection observed at fetal fractions ≥ 6%. However, these findings should be interpreted as evidence of analytical feasibility rather than clinical validation, as synthetic reference materials cannot fully reproduce the biological complexity of circulating cell-free DNA in clinical pregnancies. Importantly, the present study was designed to compare complete workflow configurations rather than isolate sequencing depth as a single experimental variable. Consequently, the observed differences likely reflect the combined effects of sequencing depth, baseline construction, bioinformatic processing, and workflow-specific technical characteristics. Overall, the results support the concept that optimized workflow design, combining adequate sequencing depth with robust baseline development and normalization strategies, may improve the analytical performance of NIPT and facilitate the detection of subchromosomal abnormalities. Nevertheless, the present study should be regarded as a preliminary analytical feasibility study rather than a clinical validation study for small CNV detection. Larger prospective investigations including clinically confirmed CNV cases, systematic diagnostic confirmation, and controlled sequencing-depth analyses will be required to establish the true clinical performance, diagnostic accuracy, and operational thresholds of this approach in routine prenatal screening.

## Figures and Tables

**Table 1:** Comparison between Vazyme and Illumina workflows: performance and outcome metrics.

METRICS	VAZYME	ILLUMINA
Female fetuses	333	333
Male fetuses	357	357
Twin pregnancies	6	6
Invalid sample	4	18
Mean reads (million)	17.97	7.32
Mean fetal fraction (%)	9	9
Autosomal aneuploidies	7	11
Low fetal fraction (<4%)	11	11
Sex discordance	0	0
Aneuploidy discordance	0	3
Mean gestational age	12 + 4	12 + 4

**Table 2:** For each case, sample type, clinical risk category, fetal sex, fetal fraction (FF), and anomaly classification, detected by the Illumina and Vazyme workflows, were reported.

	*Vazyme*				*Illumina*			
Sample_ID	Sample_type	Risk	Fetal Gender	FF	Class_sx	Class_auto	Anomaly_description	FF
1	Singleton	T21	male	12%	XY	ANOMALY DETECTED	DETECTED: +21	16%
2	Singleton	T21	female	11%	XX	ANOMALY DETECTED	DETECTED: +21	12%
3	Singleton	Low	female	5%	XX	ANOMALY DETECTED	DETECTED: +21	3%
4	Singleton	T22	male	3%	XY	ANOMALY DETECTED	DETECTED: +22	4%
5	Singleton	T22	male	8%	XY	ANOMALY DETECTED	DETECTED: +22	7%
6	Twin	Low	male	11%	CHR_Y PRESENT	ANOMALY DETECTED	DETECTED: +7	12%
7	Singleton	T18	female	9%	XX	ANOMALY DETECTED	DETECTED: +18	9%
8	Singleton	T18	female	7%	XX	ANOMALY DETECTED	DETECTED: +18	5%
9	Singleton	T8	female	7%	XX	ANOMALY DETECTED	DETECTED: +8	7%
10	Singleton	Low	female	8%	XX	ANOMALY DETECTED	DETECTED: del(7)(q21.3q31.33)	9%
11	Singleton	T9, T18	male	9%	XY	ANOMALY DETECTED	DETECTED: −1; +2; −3; −4; −7; −8; +9; −10; −12; −13; −14; +15; +18; −20; +21	12%

**Table 3:** Discordant cases between the Illumina and Vazyme workflows. For each sample, fetal sex, fetal fraction (FF), and selected statistical metrics (LLR, MLLR, MR, TSTAT) are reported.

Sample_ID	*Vazyme*				*Illumina*			
6	Twin	Chr Y present	11% 25.2 M	WT	CHR Y PRESENT	DETECTED: +7	12% 12.9 M	LLR_T: 20 MLLR_T: 25 MR: 0.17 TSTAT: 8.3
3	Singleton	female	5% 32.1 M	WT	XX	DETECTED: +21	3% 8.2 M	LLR_T: 2.7 MLLR_T: 2.7 MR:1 TSTAT: 3.6
10	Singleton	female	8% 49.8 M	WT	XX	DETECTED: del(7)(q21.3q31.33)	9% 20.0 M	LLR_M: 33 MLLR_M: 37 MR: 0.4 TSTAT: −7.9 (26.8 Mb)

Abbreviations: LLR (Log Likelihood Ratio), MLLR (Mosaic Log Likelihood Ratio), TSTAT (T-statistic for long-read fragments/regional signal), MR (Mosaic Ratio), M (million reads).

**Table 4:** Performance of 22q11.2 microdeletion detection across different fetal-fraction equivalents in a 24-plex NIPT run.

FF Group	Sample ID	CNV Call	Z-Score	Overlap (%)	Cytoband	Genomic Coordinates (hg19)	CNV Size (Mb)
Negative	test1	None	–	–	–	–	–
	test2	None	–	–	–	–	–
	test3	None	–	–	–	–	–
	test4	None	–	–	–	–	–
	test5	None	–	–	–	–	–
	test6	None	–	–	–	–	–
	test7	None	–	–	–	–	–
	test8	None	–	–	–	–	–
	test9	None	–	–	–	–	–
	test10	None	–	–	–	–	–
	test11	None	–	–	–	–	–
	test12	None	–	–	–	–	–
12% FF	test13	del22q11.2	−12.59	100.00	q11.21	18,500,001–21,500,000	3.00
	test14	del22q11.2	−11.81	100.00	q11.21	19,000,001–21,500,000	2.50
	test15	del22q11.2	−12.11	100.00	q11.21	19,000,001–22,000,000	3.00
9% FF	test16	del22q11.2	−10.99	65.50	q11.21	19,500,001–21,100,000	1.60
	test17	del22q11.2	−11.46	100.00	q11.21	19,000,001–21,500,000	2.50
	test18	del22q11.2	−11.36	100.00	q11.21	19,000,001–21,500,000	2.50
6% FF	test19	del22q11.2	−10.33	96.31	q11.21	19,100,001–22,000,000	2.90
	test20	del22q11.2	−9.17	96.31	q11.21	19,100,001–22,100,000	3.00
	test21	del22q11.2	−9.12	85.57	q11.21	19,000,001–21,100,000	2.10
2% FF	test22	None	–	–	–	–	–
	test23	None	–	–	–	–	–
	test24	del22q11.2	−7.37	79.93	q11.21	19,500,001–22,000,000	2.50

**Table 5:** Summary of clinical outcomes in the 300-sample cohort analyzed with the NIPT workflow.

Parameter	Value
Total samples (*n*)	300
Female fetuses	161
Male fetuses	131
Invalid samples	5
Twin pregnancies	3
High-risk result—Trisomy 13 (T13)	1 (grey zone)
High-risk result—Trisomy 18 (T18)	1
High-risk result—Trisomy 21 (T21)	3
High-risk result—Sex Chromosome Aneuploidy (SCA)	1
High-risk result—Microdeletion/Microduplication (MMS)	1 (false positive)
High-risk result—Rare Autosomal Aneuploidy (RAA)	1

**Table 6:** List of high-risk NIPT cases with corresponding Z-scores and additional metrics. Grey-zone and false-positive results are indicated.

Case	Condition	Z-Score	Additional Metrics	Interpretation
1	Trisomy 15 (T15)	11.851	—	High risk
2	Trisomy 13 (T13)	3.4178	—	Grey zone
3	Trisomy 18 (T18)	7.4838	—	High risk
4	Trisomy 21 (T21)	20.687	—	High risk
5	Trisomy 21 (T21)	4.679	—	High risk
6	Trisomy 21 (T21)	11.3326	—	High risk
7	Monosomy X (X0)	—	Gap FFxy = 12.8	High risk
8	22q11.2 deletion syndrome	−7.44	FF = −3.02; Overlap = 100%	False positive

## Data Availability

Protocols and identified, aggregated data that underlie the results reported in this article are available for non-commercial scientific purposes upon reasonable request from the corresponding author. Due to ethical and privacy restrictions associated with prenatal genomic data, individual-level sequencing data are not publicly available.

## Supplementary Materials

The following supporting information can be downloaded at: https://www.mdpi.com/article/10.3390/diagnostics16142138/s1, Table S1: Comparative Table of Workflows.
