# Supplementary material for: Toward Small CNV Detection in NIPT: A Preliminary Analytical Evaluation of Sequencing Depth and Workflow Configuration
Source: Diagnostics (Basel). 2026 Jul 8;16(14):2138. doi: 10.3390/diagnostics16142138 (PMC13409676; doi:10.3390/diagnostics16142138)
Supplement: Supplementary file 1 [file diagnostics-16-02138-s001.zip › diagnostics-4357564-supplementary.pdf]

Workflow Comparison

Table S1: Comparative Table of Workflows

Feature	Vazyme Workflow (24-plex)	Illumina VeriSeq Workflow (48-plex)
Library kit	VAHTS® NIPT Library Prep Kit	VeriSeq NIPT Solution v2
CE-IVD marking	RUO	CE-IVD
cfDNA input	5–20 ng	5–10 ng
Indexing	Single index N805-PB2	Proprietary VeriSeq indexes
Pool size	24 samples	48 samples
Sequencing platform	NextSeq 550Dx	NextSeq 550Dx
Chemistry	SE 1 × 75 bp	PE 2 × 75 bp
Target reads/sample	>20 M	8–10 M
Main applications	Aneuploidies + CNVs <3 Mb	Aneuploidies

The choice of the Vazyme workflow in a 24-plex configuration was driven by both operational and analytical considerations. Reducing the number of samples per run enables increased sequencing depth per sample, improving chromosomal metric stability and increasing statistical power for the detection of subchromosomal copy number variations (CNVs). Higher read depth reduces stochastic variability in bin-level coverage and improves z-score robustness, particularly in samples with low fetal fraction.
